# KLF8 knockdown suppresses proliferation and invasion in human osteosarcoma cells

**DOI:** 10.3892/mmr.2014.2027

**Published:** 2014-03-07

**Authors:** FENG LIN, ZAN SHEN, LI-NA TANG, SHUI-ER ZHENG, YUAN-JUE SUN, DA-LIU MIN, YANG YAO

**Affiliations:** 1 Graduate School of Medicine, Soochow University, Suzhou, Jiangsu 215006, P.R. China; 2 Department of Oncology, Shanghai Jiaotong University Affiliated Sixth People’s Hospital, Shanghai 200233, P.R. China

**Keywords:** krüppel-like factor 8, osteosarcoma, proliferation, migration

## Abstract

Krüppel-like factor 8 (KLF8) is a transcription factor that is important in the regulation of the cell cycle and has a critical role in oncogenic transformation and epithelial to mesenchymal transition (EMT). EMT is a key process in tumor metastasis. Although overexpression of KLF8 has been observed in a variety of human tumor types, the role of KLF8 in human osteosarcoma is yet to be elucidated. The present study aimed to investigate the biological impact of KLF8 on Saos-2 osteosarcoma cells. KLF8 gene expression was knocked down *in vitro* using a lentivirus-mediated small interfering (si)RNA method. Cell proliferation and cell cycle distribution were evaluated using 3-(4,5)-dimethylthiahiazo(-z-yl)-3,5-di-phenytetrazoliumromide and colony formation assays, and flow cytometry, respectively. Cell invasion was analyzed using a Transwell^®^ invasion assay. Knockdown of KLF8 was found to significantly inhibit proliferation and invasion in osteosarcoma cells. These data suggest that KLF8 may exhibit an important role in osteosarcoma tumorigenesis and that KLF8 may be a potential therapeutic target for the treatment of osteosarcoma.

## Introduction

Osteosarcoma is an aggressive type of malignant cancer, which develops from primitive transformed cells of mesenchymal origin. Osteosarcoma is the most common histological form of primary bone cancer (1). Despite recent improvements in the long term prognosis of patients with osteosarcoma, the identification of novel therapeutic molecular targets and therapeutic strategies for the prevention and treatment of osteosarcoma are required. The development of osteosarcoma involves the accumulation of multiple genetic and epigenetic changes in critical genes that control cell proliferation and migration (2). Understanding these mechanisms of proliferative alteration and advanced metastasis in osteosarcoma is important for the treatment of the disease.

Krüppel-like factor 8 (KLF8) is a ubiquitously expressed Krüppel-like transcription factor. Members of the KLF family contain a C-terminal DNA-binding domain with three Krüppel-like zinc fingers (3,4). Several members of the KLF family have been shown to have diverse functions in various cell types (5). KLF8 has been reported to be a critical mediator of oncogenic transformation, EMT and invasion (6–9). Increased KLF8 expression has been observed in several cancer tissues compared with that in normal tissues (10,11). Furthermore, developments in functional genomics have led to a greater focus on the biological function of KLF8 and its interaction with genes and proteins. Recent research has identified poly (ADP-ribose) polymerase 1 and matrix metallopeptidase 9 as novel KLF8-interacting and -regulating proteins (12,13). In addition, KLF8 has been identified to be a novel Wnt/beta-catenin signaling target gene and regulator (14).

The role of KLF8 in osteosarcoma is yet to be elucidated. In the present study, lentivirus-mediated siRNA was employed to knockdown KLF8 expression in the Saos-2 human osteosarcoma cell line. The effects of KLF8 on osteosarcoma cell growth and invasion were subsequently investigated.

## Materials and methods

### Materials

The Saos-2 cell line was purchased from the Institute of Biochemistry and Cell Biology (Shanghai, China). KLF8 and GAPDH primers were synthesized by Applied Biosystems (Carlsbad, CA, USA). All antibodies were purchased from Santa Cruz Biotechnology, Inc. (Dallas, TX, USA) unless stated otherwise.

### Drugs and reagents

3-(4,5)-dimethylthiahiazo(-z-yl)-3,5-di-phenytetrazoliumromide (MTT) was purchased from Shanghai Dingguo Biological Technology Co., Ltd. (Shanghai, China). Dulbecco’s modified Eagle’s medium (DMEM) and fetal bovine serum (FBS) were purchased from Thermo Fisher Scientific Inc. (Waltham, MA, USA). TRIzol^®^ Reagent and Lipofectamine^®^ 2000 were purchased from Invitrogen Life Technologies (Carlsbad, CA, USA). M-MLV Reverse Transcriptase was purchased from Promega Corporation (Madison, WI, USA) and SYBR^®^-Green PCR Master mix was purchased from Takara Bio Inc. (Shiga, Japan). A Cell cycle analysis kit and an apoptosis kit were purchased from Nanjing KeyGen Biotech., Co., Ltd. (Nanjing, China). An ECL-PLUS™ kit was purchased from GE Healthcare (Piscataway, NJ, USA).

### Cell culture

Saos-2 cells were cultured in DMEM supplemented with 10% heat-inactivated FBS, 100 U/ml penicillin and 100 μg/ml streptomycin. Cells were incubated in a humidified atmosphere containing 5% CO_2_ at 3°C.

### Lentivirus packaging and infection

Small interfering (si)RNA targeting the KLF8 gene (CAGCACTGTTTAATGACAT) and negative control siRNA (TTCTCCGAACGTGTCACGT) were cloned into a pGCSIL-green fluorescent protein (GFP) vector (Shanghai GeneChem Co., Ltd., Shanghai, China). The siRNA plasmids were transfected into 293T cells, together with two lentiviral packaging plasmids (pHelper1.0 and pHelper2.0; Shanghai Gene ChemCo., Ltd.) to generate a lentivirus. After three days of incubation, the culture medium containing the recombinant virus was collected and concentrated using Centricon^®^-plus-20 (Millipore, Billerica, CA, USA). For lentiviral infection, 5×10^4^/well Saos-2 cells were incubated with the KLF8 siRNA-expressing lentivirus and non-silencing control lentivirus [multiplicity of infection (MOI)=20] for 24 h. The culture medium was then replaced.

### Quantitative polymerase chain reaction (qPCR) analysis

Lentiviral transduction efficiency was validated using qPCR analysis after transduction for 72 h. Total RNA was extracted using TRIzol reagent and reverse transcribed using M-MLV Reverse Transcriptase according to the manufacturer’s instructions. The resulting complementary (c)DNA was used for qPCR analysis using SYBR-Green PCR Master mix. qPCR analysis was performed in triplicate using the TP800 qPCR System (Takara Bio Inc.). Target gene expression was normalized to that of the endogenous control GAPDH. The relative quantitative expression of the target gene compared with GAPDH was expressed as 2^−(Ct-Cc)^ (Ct and Cc represent the mean threshold cycle differences following normalization to GAPDH). The qPCR primer sequences were as follows: Forward: 5′-TTCAGAAGGTGGCTCAATGC-3′ and reverse: 5′-GGAGTGTTGGAGAAGTCATATTAC-3′ for KLF8; and forward: 5′-TGACTTCAACAGCGACACCCA-3′ and reverse: 5′-GGAGTGTTGGAGAAGTCATATTAC-3′ for GAPDH.

### Western blot analysis

Lentiviral transduction efficiency was validated using western blot analysis after transduction for four days. In brief, cells were harvested following four days of infection and treated with buffer containing 50 mM Tris-HCl (pH 7.5), 150 mM NaCl, 25 mM β-glycerophosphate, 50 mM NaF, 1 mM Na_3_VO_4_, 1% Triton X-100, 10% glycerol and protease inhibitors (1 mM phenylmethylsufonyl fluoride and 1 mg/ml aprotinin, pepstatin A, and leupeptin). Cell lysates were separated using 12% SDS-PAGE and transferred onto polyvinylidene fluoride membranes (Millipore). Subsequent to blocking, membranes were incubated in milk containing mouse anti-KLF8 monoclonal antibodies (dilution, 1:1,000; Abcam, Cambridge, MA, USA). Western blots were developed using horseradish peroxidase-conjugated goat anti-mouse immunoglobulin G (dilution 1:5,000) and the immunoreactive bands were detected using an enhanced chemiluminescence reagent (Millipore). Endogenous GAPDH was used as an internal control.

### Cell proliferation assay

Following confirmation of the transduction efficiency, cells were seeded onto 96-well plates at a density of 2,000 cells/well on day zero for the MTT assay. Cell growth was measured daily until day five. A volume of 20 μl MTT solution (5 mg/ml) was added into each well. Following incubation for 4 h at 3°C, 150 μl dimethylsulfoxide was added to dissolve the crystals. After incubation for 10 min at room temperature, the absorbance was read at 490 nm on the Shimadzu UV-1603 spectrophotometer (Shimadzu Corp., Kyoto, Japan).

### Colony forming assay

To determine the long-term inhibitory effect of the lentivirus, Saos-2 cells were cultured in six-well plates at a density of 200 cells/well and were treated with KLF8 siRNA lentivirus (RNAi^+^) or non-silencing siRNA lentivirus (RNAi^−^). Cells were incubated at 37°C in air with 5% CO_2_ and the medium was replaced every three days. After ten days, colonies were stained with Giemsa and the number of colonies containing >50 cells were counted in each well.

### Fluorescence-activated cell sorting (FACS) cell cycle analysis

Saos-2 cells infected with the KLF8 siRNA-expressing lentivirus and negative control lentivirus were collected three days following infection. For cell cycle analysis, cells were collected and cultured in 6-cm dishes until they reached 80% confluency. A total of 1×10^6^ cells were harvested and fixed in 70% ethanol for 1 h. Subsequent to three washes, cells were treated with 50 μl/ml propidium iodide (PI) solution (Sigma-Aldrich, St. Louis, MO, USA) and 100 μl/ml RNase in phosphate-buffered saline for 15 min at room temperature in the dark. Flow cytometric analysis was then performed using a BD FACSCalibur flow cytometer (BD Biosciences, San Jose, CA, USA).

### Transwell^®^ invasion assay

An *in vitro* cell invasion assay was performed using a Transwell unit (8-μm pore size) with polyvinylpyrrolidone-free polycarbonate filters coated with 500 μg/ml BD Matrigel™ Basement Membrane Matrix (BD Biosciences) placed in 24-well Transwell chambers. Saos-2 cells were placed in the upper compartment of the chamber and cells were allowed to attach for 8 h. Cells were then incubated in FBS-free medium for 36 h at 37°C in 5% CO_2_. DMEM containing 10% FBS was placed in the lower compartment of the chamber. Following incubation for 24 h, the filter inserts were removed from the wells and the cells on the upper side of the filter were removed using cotton swabs. The cells that had invaded the lower surface of the membrane were fixed using methanol and stained with 0.5% crystal violet for 10 min. Cells that had migrated to the lower side of the filter were scored visually in five random fields using a light microscope (10× objective lens; Nikon, Tokyo, Japan). The number of cells from three filters was then averaged. In addition, the invaded cells were lysed and quantified at 570 nm using the Shimadzu UV-1603 spectrophotometer. The experiments were repeated three times with three wells for each treatment.

### Statistical analysis

Statistical analyses were performed using GraphPad Prism 5.0 software (GraphPad Software, Inc., San Diego, CA, USA). Continuous variables were compared using Student’s t-test. P<0.05 was considered to indicate a statistically significant difference.

## Results

### Lentivirus-mediated knockdown of KLF8 in Saos-2 cells

**Figure 1:**
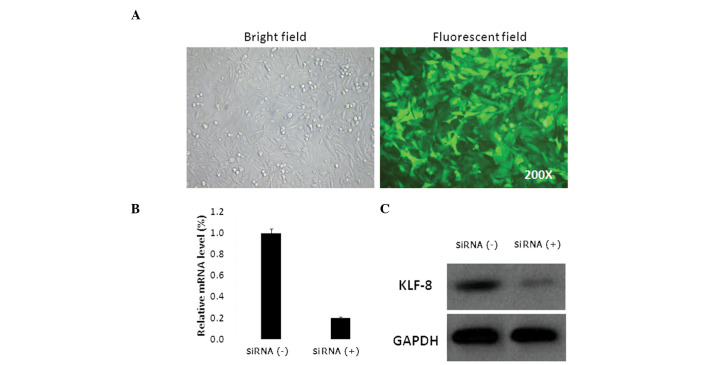
KLF8 siRNA downregulates KLF8 expression in Saos-2 osteosarcoma cells. (A) Nonsense siRNA and KLF8 siRNA-expressing lentiviral vectors were transduced into Saos-2 cells. (B) KLF8 siRNA decreased KLF8 mRNA expression in Saos-2 cells as evidenced by quantitative polymerase chain reaction analysis. Values are presented as the mean ± standard deviation of three independent experiments. (C) Lentivirus-transduced cells were lysed and subjected to western blot analysis to detect KLF-8 protein expression using anti-KLF8 and -GAPDH antibodies. siRNA, small interfering RNA; KLF8, Krüppel-like factor 8.

To determine the effect of KLF8 expression on osteosarcoma cell growth, lentiviruses expressing KLF8-specific siRNA were generated. GFP expression was observed in >90% of Saos-2 cells 72 h after lentivirus infection at an MOI=40 (Fig. 1A). qPCR analysis revealed that KLF8 mRNA expression in Saos-2 cells infected with KLF8 lentiviral siRNA was significantly decreased. (P<0.05; Fig. 1B). Western blot analysis of cell lysates extracted four days after lentiviral infection revealed that KLF8 protein expression was also decreased (Fig. 1C). These findings demonstrate that the lentivirus transduction system successfully downregulated KLF8 expression at the mRNA and protein levels compared with the Saos-2 cells infected with nonsense lentiviral siRNA.

### Saos-2 cell proliferation is inhibited by KLF8 siRNA

**Figure 2:**
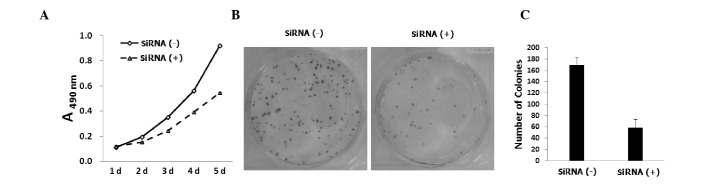
Inhibition of cell proliferation in Saos-2 cells using a KLF8 siRNA-expressing lentivirus. (A) Cell proliferation was measured using a 3-(4,5)-dimethylthiahiazo(-z-yl)-3,5-di-phenytetrazoliumromide assay at an optical density of 490 nm in Saos-2 cells infected with nonsense siRNA- and KLF8 siRNA-expressing lentiviral vectors. (B) Saos-2 cells infected with nonsense siRNA- and KLF8 siRNA-expressing lentiviral vectors were seeded in six-well plates and measured 10 days after plating. Representative images of Giemsa-stained Saos-2 cell colonies 4 days after infection. (C) Number of colonies in Saos-2 cells infected with nonsense siRNA- and KLF8 siRNA-expressing lentiviral vectors 10 days after seeding. Values are presented as the mean ± standard deviation of three independent experiments. siRNA, small interfering RNA; KLF8, Krüppel-like factor 8; A, absorbance.

To assess the effect of KLF8 knockdown on osteosarcoma cell proliferation, Saos-2 cells were infected with KLF8 siRNA-expressing lentiviral vectors and viable cells were counted using an MTT assay five days post-infection. As shown in Fig. 2A, KLF8 knockdown was found to decreased the number of Saos-2 cells compared with the control siRNA-infected cells (P<0.05). Lentiviral KLF8 siRNA-infected Saos-2 cells were analyzed using a colony forming assay. Downregulation of KLF8 was found to reduce the number of viable Saos-2 cell colonies (Fig. 2B), suggesting that the KLF8 siRNA-treated cells had a lower colony formation ability compared with the control siRNA-infected cells (P<0.05; Fig. 2C). In summary, KLF8 knockdown was observed to inhibit cell growth and colony formation in Saos-2 cells.

### KLF8 knockdown arrests Saos-2 cells in G_0_/G_1_-phase

**Figure 3:**
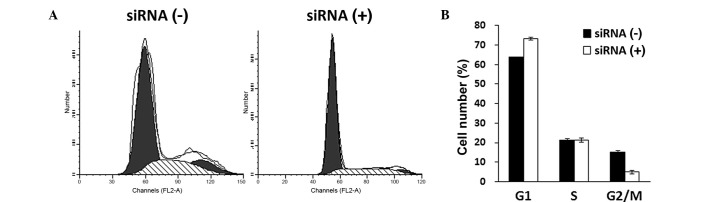
KLF8 siRNA induces cell cycle arrest in G_0_/G_1_-phase. (A) Fluorescence-activated cell sorting analysis of cell cycle phase in Saos-2 cells treated with KLF8 siRNA. (B) Cells treated with KLF8 siRNA exhibit a greater distribution of cells in G0/G1-phase. Values are presented as the mean ± standard deviation of three independent experiments. siRNA, small interfering RNA; KLF8, Krüppel-like factor 8.

Cell cycle distribution was assessed in KLF8-knockdown cells using PI staining and FACS analysis five days after lentiviral infection. Lentivirus-mediated KLF8-siRNA infection affected cell cycle distribution in Saos-2 cells, as shown in Fig. 3A. Statistical analysis revealed that KLF8 siRNA treatment arrested Saos-2 cells in G_0_/G_1_-phase of interphase of the cell cycle. Furthermore, the number of Saos-2 cells in G_2_/M phase was observed to be significantly reduced (P<0.05; Fig. 3B), indicating that DNA replication was impaired following KLF8 knockdown.

### KLF8 knockdown suppresses Saos-2 cell invasion

**Figure 4:**
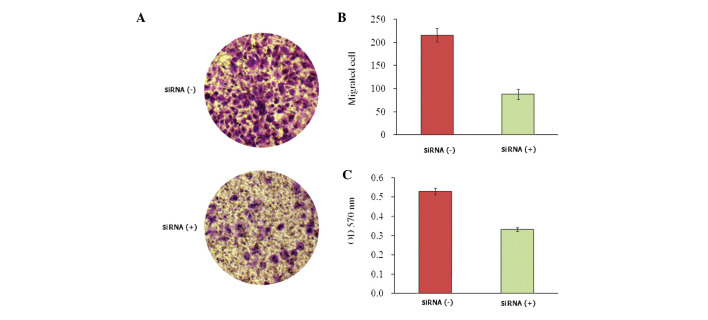
Effect of KLF8 siRNA-expressing lentivirus treatment on Saos-2 cell invasion. (A) Crystal violet staining of cells which invaded through the polycarbonate membranes in the invasion assay. (B) The average number of migrated cells observed in each group. Saos-2 cell migration was found to be significantly decreased in the KLF8 siRNA treatment group. (C) Quantitative measurement of cell invasion using a spectrophotometer. Saos-2 cell invasion was found to be significantly reduced in the KLF8 siRNA treatment group. Values are presented as the mean ± standard deviation of three independent experiments. siRNA, small interfering RNA; KLF8, Krüppel-like factor 8.

To investigate the effect of KLF8 knockdown on osteosarcoma cell invasion*,* Saos-2 cells were analyzed using a Transwell assay and crystal violet staining. As shown in Fig. 4, the invasive potential of Saos-2 cells was significantly reduced with lentivirus-mediated KLF8 siRNA treatment compared with the control group (P<0.05), indicating that KLF8 may have a role in promoting osteosarcoma cell invasion.

## Discussion

KLF8 exhibits conserved C2H2 zinc finger domains at its C-terminus through which it binds DNA, as well as a PVALS/T motif at its N-terminus through which it interacts with co-repressor C-terminal binding protein. KLF8 inhibits the expression of genes containing a CACCC element and KLF8 is overexpressed in several types of tumor cells, including gliomas (15), ovarian (7), renal (16), hepatocellular (10), gastric (6) and breast carcinoma (13) cells.

The mechanism underlying KLF8 activation in cancer cells is yet to be elucidated. Overexpression of KLF8 has been reported to be highly correlated with decreased E-cadherin expression, which is associated with cancer cell invasion (17). In a previous study, KLF8 expression was found to be regulated by focal adhesion kinase signaling (18). However, the function of KLF8 in human osteosarcoma remains unknown.

Cancer cells undergo malignant proliferation and have the capacity to migrate into the surrounding tissue, which is known as metastasis. In the present study, downregulation of KLF8 was found to inhibit Saos-2 cell proliferation and colony formation. It was hypothesized that alterations in cell cycle progression may be the primary mechanism involved in this inhibition of cancer cell growth. Therefore, the present study investigated the effect of KLF8-siRNA lentiviral infection on Saos-2 cell cycle arrest and its association with Saos-2 cell growth inhibition. A significant increase in G_0_/G_1_-phase cell cycle arrest was observed in the KLF8 knockdown cells and this was found to account for the inhibitory effect of KLF8 siRNA on Saos-2 cell proliferation. Moreover, Saos-2 cell invasion was observed to be suppressed following KLF8 knockdown. These findings show that KLF8 knockdown reduced survival and invasion in Saos-2 osteosarcoma cells. Therefore, lentivirus-mediated KLF8 siRNA treatment may be an effective therapeutic strategy for osteosarcoma.

Studies have revealed that numerous genes, including oncogenes and tumor suppressor genes are involved in the complex, multi-step process of tumorigenesis (19,20). Advances in bioscience have enhanced the understanding of the molecular mechanisms underlying osteosarcoma progression. There are now promising research opportunities to screen and identify molecular targets for anticancer applications. The present study identified the important role of KLF8 in osteosarcoma and its potential as a biomarker for diagnosis and therapy. The present study provides evidence that lentivirus-mediated KLF8 knockdown inhibits growth and invasion in osteosarcoma cells, suggesting that KLF8 may be a potential therapeutic biomarker for osteosarcoma. Further investigations into the molecular mechanisms underlying the regulation of cell proliferation and invasion by KLF8 are required in the future.
